# Cannabidiol Is a Novel Modulator of Bacterial Membrane Vesicles

**DOI:** 10.3389/fcimb.2019.00324

**Published:** 2019-09-10

**Authors:** Uchini S. Kosgodage, Paul Matewele, Brigitte Awamaria, Igor Kraev, Purva Warde, Giulia Mastroianni, Alistair V. Nunn, Geoffrey W. Guy, Jimmy D. Bell, Jameel M. Inal, Sigrun Lange

**Affiliations:** ^1^Cellular and Molecular Immunology Research Centre, School of Human Sciences, London Metropolitan University, London, United Kingdom; ^2^School of Life, Health and Chemical Sciences, The Open University, Milton Keynes, United Kingdom; ^3^Bioscience Research Group, Extracellular Vesicle Research Unit, School of Life and Medical Sciences, University of Hertfordshire, Hatfield, United Kingdom; ^4^School of Biological and Chemical Sciences, Queen Mary University of London, London, United Kingdom; ^5^Research Centre for Optimal Health, School of Life Sciences, University of Westminster, London, United Kingdom; ^6^GW Pharmaceuticals Research, Cambridge, United Kingdom; ^7^Tissue Architecture and Regeneration Research Group, School of Life Sciences, University of Westminster, London, United Kingdom

**Keywords:** bacterial membrane vesicles (MVs), cannabidiol (CBD), antibiotic resistance, gram-negative, gram-positive, *E. coli* VCS257, *S. aureus* subsp*. aureus* Rosenbach

## Abstract

Membrane vesicles (MVs) released from bacteria participate in cell communication and host-pathogen interactions. Roles for MVs in antibiotic resistance are gaining increased attention and in this study we investigated if known anti-bacterial effects of cannabidiol (CBD), a phytocannabinoid from *Cannabis sativa*, could be in part attributed to effects on bacterial MV profile and MV release. We found that CBD is a strong inhibitor of MV release from Gram-negative bacteria (*E. coli* VCS257), while inhibitory effect on MV release from Gram-positive bacteria (*S. aureus* subsp*. aureus* Rosenbach) was negligible. When used in combination with selected antibiotics, CBD significantly increased the bactericidal action of several antibiotics in the Gram-negative bacteria. In addition, CBD increased antibiotic effects of kanamycin in the Gram-positive bacteria, without affecting MV release. CBD furthermore changed protein profiles of MVs released from *E. coli* after 1 h CBD treatment. Our findings indicate that CBD may pose as a putative adjuvant agent for tailored co-application with selected antibiotics, depending on bacterial species, to increase antibiotic activity, including via MV inhibition, and help reduce antibiotic resistance.

## Introduction

Outer membrane vesicles (OMVs) and membrane vesicles (MVs) are released from Gram-negative and Gram-positive bacteria and participate in inter-bacterial communication, including via transfer of cargo molecules (Dorward and Garon, 1990; Li et al., 1998; Fulsundar et al., 2014; Jan, 2017; Toyofuku et al., 2019). MVs are released in greater abundance from Gram-negative, than Gram-positive bacteria and their production seems crucial for bacterial survival and forms part of the stress response (McBroom and Kuehn, 2007; Macdonald and Kuehn, 2013; Jan, 2017). Gram-negative bacteria generate, in addition to common one-bilayer vesicles (OMV), also double-bilayer vesicles (O-IMVs), and in some stress conditions other types of MVs (Pérez-Cruz et al., 2016) and therefore we will use the umbrella term “membrane vesicles” (MVs) hereafter. MVs are important in biofilm formation and dissemination of toxins in the host (Wang et al., 2015; Cooke et al., 2019). MVs participate in host-pathogen interactions (Gurung et al., 2011; Koeppen et al., 2016; Bitto et al., 2017, 2018; Codemo et al., 2018; Turner et al., 2018; Cecil et al., 2019) and may also be involved in antibiotic resistance, for instance by protecting biofilms from antibiotics via increased vesiculation (Manning and Kuehn, 2011). Furthermore, MVs from *Porphyromonas gingivalis* have been linked to metabolic remodeling in the host (Fleetwood et al., 2017), while MVs from *Neisseria gonorrhoeae* have been shown to target host mitochondria and to induce macrophage death (Deo et al., 2018). Besides roles for cellular and bacterial communication, the use of MVs as nano-carriers for various compounds, including for antibiotic and vaccine delivery, has also raised considerable interest in the research community (Gnopo et al., 2017; Rüter et al., 2018; Tan et al., 2018; Wang et al., 2018).

The regulation of bacterial MV biogenesis and release may therefore be of great importance, both in relation to inter-bacterial communication, including biofilm formation, their host interactions as commensals, as well as in host-pathogen interactions and in antibiotic resistance.

Cannabidiol (CBD) is a phytocannabinoid from *Cannabis sativa* with anti-inflammatory (Martin-Moreno et al., 2011), anti-cancerous (Pisanti et al., 2017; Kosgodage et al., 2018) and anti-bacterial activity (Hernández-Cervantes et al., 2017). While immunoregulatory roles for cannabinoids have been reported in infectious disease (reviewed in Hernández-Cervantes et al., 2017), and *C. sativa* has been identified as a natural product with a capability of controlling bacterial infections, including a strong anti-bacterial activity against antibiotic resistant strains (Appendino et al., 2008), a link between CBD and bacterial MV release has hitherto not been investigated.

As our recent work identified CBD as a potent inhibitor of extracellular vesicle (EV) release in eukaryotes (Kosgodage et al., 2018; Gavinho et al., 2019), we sought to investigate whether CBD may work via phylogenetically conserved pathways, involving bacterial MV release from bacteria. As we, and other groups, have previously shown that cancer cells can be sensitized to chemotherapeutic agents via various EV-inhibitors (Jorfi et al., 2015; Koch et al., 2016; Muralidharan-Chari et al., 2016; Kosgodage et al., 2017), including CBD (Kosgodage et al., 2018, 2019), we sought to establish whether in bacteria, similar putative MV modulatory effects could be utilized to sensitize bacteria to antibiotics.

## Materials and Methods

### MV Isolation From *E. coli* VCS257 and *S. aureus* subsp*. aureus* Rosenbach

*E. coli* (VCS257, Agilent, La Jolla, CA) and *S. aureus* subsp*. aureus* Rosenbach (ATCC 29247, USA) static cultures were grown in Luria-Bertani (LB) broth for 24 h at 37°C. The growth phase before vesicle isolation was exponential; the volume of the cultures was 20 ml. For MV isolation, ultracentrifugation and nanoparticle tracking analysis (NTA) were used based on previously established methods by other groups (McCaig et al., 2013; Klimentova and Stulik, 2015; Roier et al., 2016).

*E. coli* and *S. aureus* cultures were maintained by plating on Mueller-Hinton agar plates and weekly sub-culturing was performed according to previously established methods (Iqbal et al., 2013).

Before MV isolation, all bacterial growth medium (LB broth) was pre-treated before use by ultracentrifugation at 100,000 g for 24 h to ensure minimum contamination with extracellular vesicles (EVs) from the medium (Kosgodage et al., 2017).

For MV isolation, bacteria were grown in EV-free medium (as described above) for 24 h at 37°C, the culture medium was collected and centrifuged once at 400 g for 10 min for removal of cells, followed by centrifugation at 4,000 g for 1 h at 4°C to remove cell debris. The resultant supernatant was then centrifuged for 1 h at 100,000 g at 4°C and the isolated MV pellet was resuspended in Dulbecco's phosphate buffered saline (DPBS; ultracentrifuged and sterile filtered using a 0.22 μm filter) and centrifuged again at 100,000 *g* for 1 h at 4°C. The resulting MV pellet was sterile filtered (0.45 μm) once and then resuspended in sterile filtered DPBS. The quantitative yield of vesicles was ~6.5 × 10^9^ MVs per liter of culture. The isolated MV pellets were then either used immediately, or stored at −80°C for further experiments.

### Transmission Electron Microscopy (TEM) Imaging of Bacterial MVs

A suspension of isolated MVs (1.4 × 10^8^ MVs/ml) was used for TEM imaging. MV samples (10 μL) were applied to mesh copper grids, prepared with glow discharged carbon support films, and incubated for 2 min. The grids were then washed five times with 50 μl of 1 % aqueous uranyl acetate. Grids were left to dry for 5 min before being viewed. Micrographs were taken with a JEOL JEM 1230 transmission electron microscope (JEOL, Japan) operated at 80 kV at a magnification of 80,000 to 100,000. Digital images were recorded using a Morada CCD camera (EMSIS, Germany) and processed via iTEM (EMSIS).

### Western Blotting

Protein was isolated from MV pellets using Bacterial Protein Extraction Reagent (B-PER, ThermoFisher Scientific, U.K.), pipetting gently and shaking the pellets on ice for 2 h, where after samples were centrifuged at 16,000 g at 4°C for 20 min and the resulting supernatant collected for protein analysis. Samples were prepared in 2x Laemmli buffer, boiled at 95°C for 5 min, electrophoresed by SDS-PAGE on 4–20 % TGX gels (BioRad, U.K.), followed by semi-dry Western blotting. Approximately 10 μg of protein was loaded per lane and even protein transfer was assessed by Ponceau S staining (Sigma, U.K.). Blocking of membranes was performed for 1 h at room temperature (RT) in 5 % BSA in TBS-T. The membranes were then incubated with the anti-OmpC (Outer-membrane protein C antibody; orb6940, Biorbyt, U.K.; diluted 1/1000 in TBS-T) overnight at 4°C, followed by washing in TBS-T and incubation for 1 h in anti-rabbit-HRP conjugated secondary antibody at RT. Visualization was performed using ECL (Amersham, U.K.) and the UVP BioDoc-ITTM System (U.K.).

### Nanoparticle Tracking Analysis for Assessment of MV Release From *E. coli* VCS257 and *S. aureus* subsp. *aureus* Rosenbach

MVs were isolated from control and CBD-treated bacterial cultures as described above. Nanoparticle tracking analysis (NTA) was performed using the Nanosight LM10 (Malvern, U.K.), equipped with a 405 nm diode laser and a sCMOS camera. MV pellets were resuspended in equal volumes (100 μl) of DPBS before NTA analysis to ensure comparable analysis of quantification. Before application, samples were diluted 1:50 in sterile-filtered EV-free DPBS and applied at a constant flow rate, maintaining the number of particles in the field of view in the range of 20–40 with a minimum concentration of samples at 5 × 10^7^ particles/ml. Camera settings were according to the manufacturer's instructions (Malvern), five 60 s videos per sample were recorded and replicate histograms averaged. Each experiment was repeated three times.

### CBD-Mediated MV Release Inhibition in *E. coli* VCS257 and *S. aureus* subsp. *aureus* Rosenbach

*E. coli* and *S. aureus* cultures were cultivated using EV-free Müeller-Hinton broth for 24 h. An inoculate of 0.1 ml of bacteria, in a 20 ml culture volume of bacterial growth medium (Luria-Bertani (LB) broth), were grown at exponential phase overnight, as assessed by OD600. The bacterial cells were then washed using DPBS at 4,000 g for 10 min and seeded in 1.5 ml triplicates in micro centrifuge tubes. For treatment with CBD, CBD (GW research Ltd) was applied at concentrations of 1 or 5 μM and incubated with the bacterial cultures for 1 h at 37°C. Treatments were performed in triplicates, including DMSO as a control. MV isolation following CBD and control treatment was carried out using step-wise centrifugation and ultracentrifugation as before. Changes in MV release were assessed by quantifying numbers of MVs by NTA analysis as described above, with each experiment repeated three times. Cell viability was assessed before the start of every experiment and after treatment with CBD compared to controls determined by colony forming unit (CFU) measurement.

### Disc Diffusion Test for Assessment of CBD-Mediated Enhancement of Antibiotic Treatment

Discs were impregnated with the following antibiotics (all from Sigma-Aldrich): colistin (10 μg/ml), rifampicin (15 μg/ml), erythromycin (50 μg/ml), kanamycin (1,000 μg/ml) and vancomycin (5 μg/ml). Concentration of the antibiotics used was based on previously published and established MIC values (Maclayton et al., 2006; Moskowitz et al., 2010; Kshetry et al., 2016; Rojas et al., 2017; Goldstein et al., 2018). *E. coli* and *S. aureus* agar plates were prepared for the disc diffusion test (Iqbal et al., 2013) by soaking a sterile paper disc in 5 μM CBD and placing it in the middle of the agar plate, while the impregnated antibiotic discs were placed equidistant to the CBD disc. Zones of inhibition were assessed after 24 h using the Kirby-Bauer test.

### Proteomic Analysis of MVs Released From CBD Treated and Control Untreated *E. coli* VCS257

To assess differences in *E. coli* VCS257 MV protein composition in response to CBD treatment, MVs were isolated as before, after 1 h treatment with 1 μM or 5 μM CBD treatment or control untreated, respectively. MVs were assessed by SDS-PAGE (using 4–20 % gradient TGX gels, BioRad, U.K.) and silver staining using the BioRad Silver Stain Plus Kit (1610449, BioRad, U.K.), according to the manufacturer's instructions (BioRad). For assessment of proteomic changes, MVs were subjected to liquid chromatography-mass spectrometry (LC-MS/MS) analysis. MVs from CBD treated, vs. non-treated *E. coli* were run 1 cm into a SDS-PAGE gel and the whole protein lysate cut out as one band, whereafter it was processed for proteomic analysis (carried out by Cambridge Proteomics, U.K.). Peak list files were submitted to Mascot (in-house, Cambridge Center for Proteomics) using the following database: Uniprot_Escherichia_coli_20180613 (4324 sequences; 1357163 residues).

### Statistical Analysis

Histograms and graphs were prepared and statistical analysis was performed using GraphPad Prism version 8 (GraphPad Software, San Diego, U.S.A.). One-way ANOVA and Student's *t*-test analysis were performed, followed by Tukey's *post-hoc* analysis. Histograms represent mean of data, with error bars representing standard error of mean (SEM). Significant differences were considered as *p* ≤ 0.05.

## Results

### Characterization of MVs From *E. coli* VCS257 and *S. aureus* subsp. *aureus* Rosenbach

**Figure 1:**
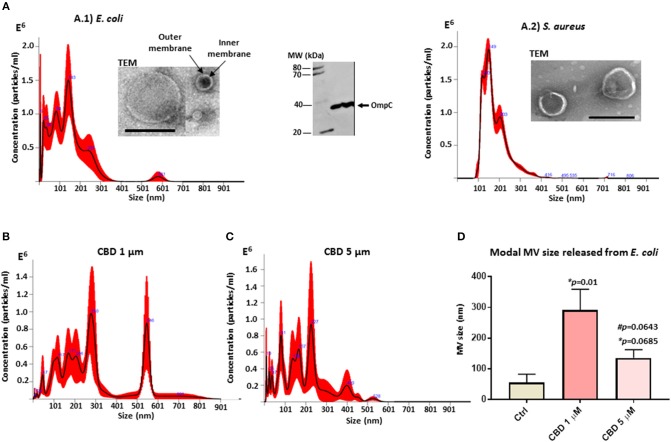
Bacterial MV profile under standard conditions and after CBD treatment. **(A)** MVs released from untreated *E. coli* VCS257 **(A.1)** and *S. aureus* subsp. *aureus* Rosenbach **(A.2)**, shown by NTA analysis (Nanosight); Transmission electron microscopy (TEM, scale bar = 200 nm) and Western blotting with the MV-specific marker OmpC. **(B)** NTA analysis showing MV release from *E. coli* after 1 h CBD treatment (1 μM). **(C)** NTA analysis showing MV release from *E. coli* after 1 h CBD treatment (5 μM). **(D)** Modal size of MVs released from *E. coli* under normal culture conditions compared to CBD treatment. Error bars indicate SEM; *p represents *p*-values compared to control (ctrl) while #*p* represents *p*-values compared to 1 μM CBD treatment.

Isolated MVs were assessed by morphology using transmission electron microscopy (TEM), revealing a poly-dispersed population in the size range of mainly 20–230 nm in diameter for *E. coli*, including MVs showing inner and outer membranes (Figure 1A.1), and characteristic one layer membranes for *S. aureus* MVs, which were in the 37–300 nm range (Figure 1A.2). Nanoparticle tracking analysis (NTA) verified that the majority of the vesicle population fell in a similar size range under standard culture conditions (mode 143.3 nm; SD ± 72.3 nm for *E. coli* (Figure 1A.1) and 141.4 nm; SD ± 7.3 nm for *S. aureus* (Figure 1A.2). Furthermore, Western blotting showed positive for the MV specific marker OmpC (Figure 1A).

### Effects of CBD on Membrane Vesicle Release From *E. coli* VCS257 and *S. aureus* subsp. *aureus* Rosenbach

CBD changed the MV release profile from *E. coli* compared to control treatment (Figures 1B–D). Modal size of MVs released from *E. coli* was significantly increased (*p* = 0.01) after 1 μM CBD treatment, compared to control treated cells, while 5 μM CBD treatment did not have statistically significant effects on MV size (*p* = 0.0685). Effects on modal size of vesicles released from *E. coli* between the two doses of CBD was also not statistically significant (*p* = 0.0643; Figure 1D).

**Figure 2:**
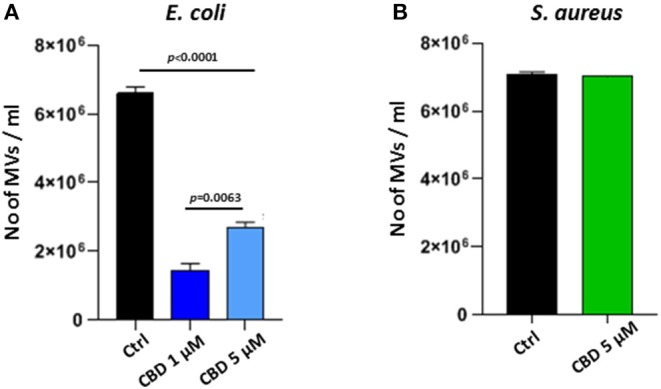
CBD affects MV-release from the Gram-negative bacteria *E. coli* VCS257 but not Gram-positive *S. aureus* subsp. *aureus* Rosenbach. **(A)** MV release from *E. coli* was significantly reduced after CBD treatment, with lower dose of CBD being more effective (*p* = 0.0063). **(B)** MV release from *S. aureus* was not significantly affected by CBD treatment. Exact *p*-values are shown.

CBD had a significant inhibitory effect (*p* < 0.0001) on total MV release from *E. coli* VCS257 at both concentrations tested (1 and 5 μM, respectively; Figure 2A). In addition, the lower dose of CBD (1 μM) had stronger MV-inhibitory effects (73 % reduction, *p* < 0.0001) than 5 μM CBD (54 % reduction, *p* < 0.0001; Figure 2A) and resulted in a markedly increased peak at 500 nm (Figure 1B), which otherwise was negligible in the control (Figure 1A) and 5 μM CBD (Figure 1C) treated *E. coli*.

Effects of CBD on *E. coli* VCS257 MVs was furthermore assessed by TEM, verifying the presence of fewer vesicles per field and showing some change in vesicle size and morphology after CBD (Supplementary Figures 2A–C).

Contrary to what was observed for the Gram-negative *E. coli*, CBD treatment (5 μM) had no significant effect on MV release from the Gram-positive bacterium *S. aureus* subsp. *Aureus* Rosenbach (*p* > 0.1; Figure 2B).

### Effects of CBD on Bacterial Viability of *E. coli* VCS257 and *S. aureus* subsp. *aureus* Rosenbach

CBD had negligible effect on *E. coli* cell viability after 24 h incubation with the lower 1 μM dose, while an 11 % (*p* = 0.0161) reduction in cell viability was observed in response to 5 μM CBD, but no significant effect was observed on *S. aureus* cell viability, as assessed by disk diffusion test (Supplementary Figure 1).

### CBD Treatment Affects Antibiotic Sensitivity in *E. coli* VCS257

**Figure 3:**
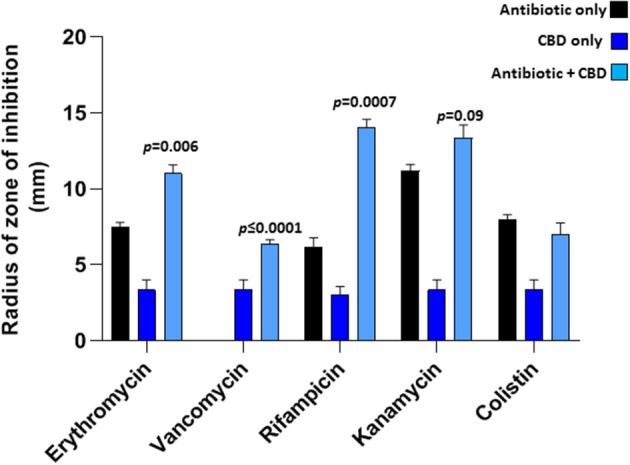
CBD sensitizes Gram-negative bacteria *E. coli* VCS257 to selected antibiotics. Combinatory treatment of CBD with a range of antibiotics (24 h treatment) showed enhanced CBD-mediated antibacterial effects on *E. coli* VCS257, as assessed by increased radius of zone around the diffusion disks. CBD was most effective in combination with rifampicin (*p* = 0.0007), vancomycin (*p* ≤ 0.0001) and erythromycin (*p* = 0.006). CBD in isolation also had bactericidal effects on *E. coli*, while combinatory treatment with the antibiotics was most effective. Exact *p*-values are shown.

CBD treatment (5 μM), when applied in combination with a range of antibiotics tested, was found to sensitize *E. coli* VCS257 to selected antibiotics, as assessed by an increase in the radius of zone of inhibition, using the disk diffusion test (Figure 3). Significantly enhanced antibacterial effects were found for erythromycin (35 % increase; *p* = 0.006), rifampicin (50 % increase; *p* = 0.0007) and vancomycin (100 % increase; *p* < 0.0001), when combined with CBD treatment (5 μM), compared to antibiotic treatment alone. Notably, vancomycin alone did not have bactericidal effects on *E. coli*, but only in the presence of CBD. Antibacterial effects of kanamycin were increased by 18 % but this was not statistically significant compared to antibiotic alone (*p* = 0.09). Zone of inhibition with CBD treatment only was also observed in the *E. coli* plates (Figure 3), but this was significantly lower than when CBD was combined with antibiotics, except for vancomycin. The zone of inhibition for *E. coli* caused by antibiotic treatment only, vs. CBD alone, differed also significantly for erythromycin (*p* = 0.0010), vancomycin (*p* = 0.0158), rifampicin (*p* = 0.0003) and kanamycin (*p* = 0.0008), but not for colistin (*p* = 0.224). Therefore, while CBD showed some anti-bacterial activity against *E. coli* when applied in isolation, this was significantly lower than observed for the antibiotics alone (except for vancomycin which did not show antibacterial activity while CBD did). However, when applied in combination, CBD increased bactericidal effects of all antibiotics tested, except for colistin.

### CBD-Mediated Effects on Antibiotic Sensitivity in *S. aureus s*ubsp. *aureus* Rosenbach

**Figure 4:**
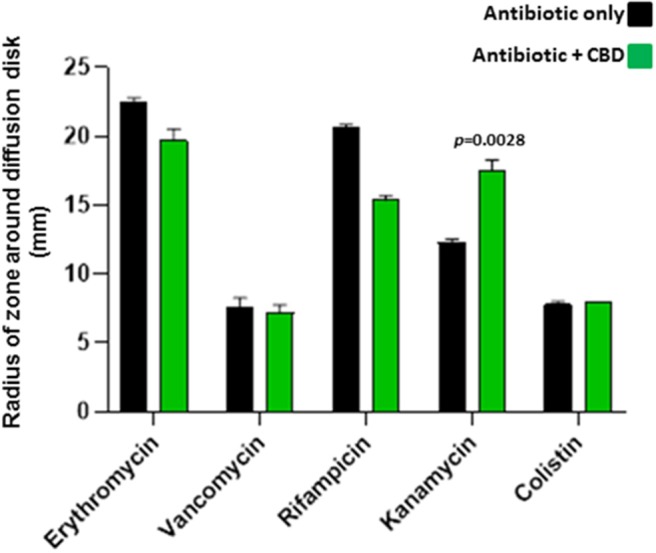
CBD sensitizes Gram-positive bacteria *S. aureus* subsp. *aureus* Rosenbach to kanamycin. Combinatory treatment of CBD with a range of antibiotics showed enhanced antibacterial effects of kanamycin only on *S. aureus*, as assessed by an increased radius of zone around the diffusion disk (*p* = 0.0028). CBD did not enhance bactericidal activity for the other antibiotics tested and reduced bactericidal effects of both erythromycin (*p* = 0.0325) and rifampicin (*p* = 0.0001). CBD application in isolation did not form a halo around the diffusion disk in the *S. aureus* plates, opposed as to what was observed in *E. coli*, and CBD treatment in isolation is therefore not included in the histogram. Exact *p*-values are shown.

When added to *S. aureus* subsp. *aureus* Rosenbach, 5 μM CBD increased the antibiotic activity of kanamycin (30 %; *p* = 0.0028), as assessed by increased radius of zone around the diffusion disk (Figure 4). CBD did not enhance anti-bacterial activity for the other antibiotics tested and reduced antibacterial effects of both erythromycin and rifampicin (*p* = 0.0325 and *p* = 0.0001, respectively). Importantly, there was no halo observed around the diffusion disk containing CBD alone in the *S. aureus* plates, indicating no bactericidal effects of CBD on this strain of *S. aureus*.

### Effects of CBD Treatment on Protein Profiles of MVs Released From *E. coli* VCS257

**Figure 5:**
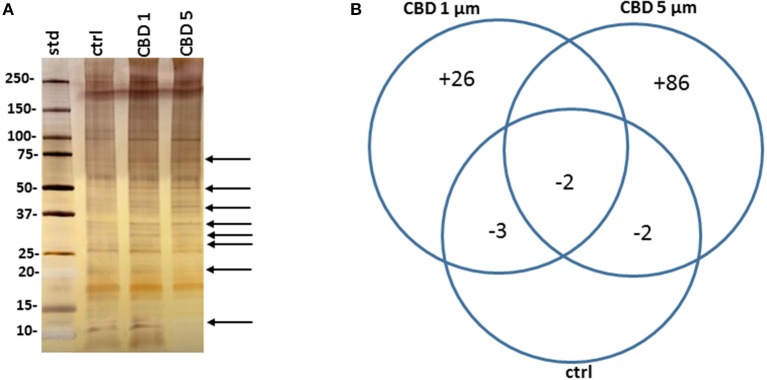
CBD affects protein composition of *E. coli* VCS257 MVs. **(A)** A SDS-PAGE silver stained gel reveals banding differences between the CBD treated and non-treated *E. coli* derived MVs (see arrows highlighting some present and absent bands). **(B)**. Venn diagram showing protein changes in MVs released from CBD treated compared to untreated control *E. coli* VCS257. Plus (“+”) indicates hits unique to MVs following CBD 1 or 5 μM treatment, respectively; minus (“–“) indicates number of proteins absent in the respective CBD treated MVs, compared to control untreated MVs. For specific protein hits see Tables 1–3.

Protein composition of MVs was assessed in MVs isolated from *E. coli* VCS257 after 1 h treatment with 1 μM and 5 μM CBD, respectively, compared to non-treated *E. coli* MVs, using SDS-PAGE silver stained gels and LC-MS/MS analysis. Silver stained gels revealed some band differences between the three conditions (Figure 5A). Proteins were further analyzed by LC-MS/MS and peak list files submitted to Mascot (in-house, Cambridge Center for Proteomics, Uniprot_Escherichia_coli_20180613). Hits are listed in Tables 1–3. Compared to untreated MVs, five protein hits were absent in MVs released from the 1 μM CBD treated *E. coli* and four protein hits were absent in MVs released from the 5 μM CBD treated *E. coli*, respectively (Table 1 and Figure 5B). When comparing the two CBD treatments, 26 protein hits were specific to the *E. coli* MVs following 1 μM CBD treatment (Table 2 and Figure 5B) while 68 protein hits were unique to the MVs released from *E. coli* treated with 5 μM CBD (Table 3 and Figure 5B).

**Table 1:** Proteins identified as present in *E. coli* VCS257 control untreated MVs only and absent in MVs from CBD treated *E. coli*.

**Protein name**	**Symbol**	**Score (*p* < 0.05)^‡^**	**CBD 1 μM**	**CBD 5 μM**
Glutamate decarboxylase alpha	P69908|DCEA_ECOLI	37	**–**	**+**
2-oxoglutarate dehydrogenase E1 component	P0AFG3|ODO1_ECOLI	36	**+**	**–**
RNA chaperone ProQ	P45577|PROQ_ECOLI	32	**–**	**+**
Uncharacterized protein YffS	P76550|YFFS_ECOLI	29	**–**	**+**
Serine transporter	P0AAD6|SDAC_ECOLI	26	**+**	**–**
Fumarate and nitrate reduction regulatory protein	P0A9E5|FNR_ECOLI	26	**–**	**–**
Uncharacterized protein YcaQ	P75843|YCAQ_ECOLI	22	**–**	**–**

*Proteins were isolated from E. coli derived MVs and analyzed by LC-MS/MS. Peak list files were submitted to Mascot (in-house, Cambridge Center for Proteomics, Uniprot_Escherichia_coli_20180613; 4324 sequences; 1357163 residues)*.

‡ Ions score is −10^*^Log(P), where P is the probability that the observed match is a random event. Individual ions scores > 19 indicated identity or extensive homology (p < 0.05). Protein scores were derived from ions scores as a non-probabilistic basis for ranking protein hits.

**Table 2:** Proteins identified as present only in MVs released from *E. coli* VCS257 following 1 h treatment with 1 μM CBD.

**Protein name**	**Symbol**	**Score (*p* < 0.05)^‡^**
Glutamate decarboxylase beta	P69910|DCEB_ECOLI	230
Tryptophan synthase alpha chain	P0A877|TRPA_ECOLI	85
2-oxoglutarate dehydrogenase E1 component	P0AFG3|ODO1_ECOLI	70
Uncharacterized protein YgaU	P0ADE6|YGAU_ECOLI	67
Spermidine/putrescine-binding periplasmic protein	P0AFK9|POTD_ECOLI	67
Serine transporter	P0AAD6|SDAC_ECOLI	57
Inorganic pyrophosphatase	P0A7A9|IPYR_ECOLI	56
Succinate dehydrogenase flavoprotein subunit	P0AC41|SDHA_ECOLI	54
NADH-quinone oxidoreductase subunit A	P0AFC3|NUOA_ECOLI	53
Periplasmic dipeptide transport protein	P23847|DPPA_ECOLI	49
Uncharacterized protein YqiC	Q46868|YQIC_ECOLI	48
Formate dehydrogenase, nitrate-inducible, major subunit	P24183|FDNG_ECOLI	47
Acyl carrier protein	P0A6A8|ACP_ECOLI	45
Maltose/maltodextrin-binding periplasmic protein	P0AEX9|MALE_ECOLI	44
Septum site-determining protein MinD	P0AEZ3|MIND_ECOLI	42
Phosphate-specific transport system accessory protein PhoU	P0A9K7|PHOU_ECOLI	40
Ribosome-associated inhibitor A	P0AD49|YFIA_ECOLI	36
DNA-binding protein H-NS	P0ACF8|HNS_ECOLI	35
RNA-binding protein Hfq	P0A6X3|HFQ_ECOLI	33
Phosphate transport system permease protein PstA	P07654|PSTA_ECOLI	32
Galactoside transport system permease protein MglC	P23200|MGLC_ECOLI	32
Sec translocon accessory complex subunit YajC	P0ADZ7|YAJC_ECOLI	31
Isoform Beta of Translation initiation factor IF-2	P0A705-2|IF2_ECOLI	30
2,3-bisphosphoglycerate-dependent phosphoglycerate mutase	P62707|GPMA_ECOLI	30
Peptidoglycan D,D-transpeptidase FtsI	P0AD68|FTSI_ECOLI	28
Inner membrane protein YjcH	P0AF54|YJCH_ECOLI	27
HTH-type transcriptional regulator GntR	P0ACP5|GNTR_ECOLI	27
Histidinol-phosphate aminotransferase	P06986|HIS8_ECOLI	26
SsrA-binding protein	P0A832|SSRP_ECOLI	25
2-dehydro-3-deoxyphosphooctonate aldolase	P0A715|KDSA_ECOLI	25
Deoxyribose-phosphate aldolase	P0A6L0|DEOC_ECOLI	25
Ribosome hibernation promoting factor	P0AFX0|HPF_ECOLI	24
Ribokinase	P0A9J6|RBSK_ECOLI	24
Probable ATP-dependent helicase l hr	P30015|LHR_ECOLI	22
Membrane-bound lytic murein transglycosylase B	P41052|MLTB_ECOLI	21
Uncharacterized protein YjaA	P09162|YJAA_ECOLI	21
Adenylate kinase	P69441|KAD_ECOLI	21
Fructose-1,6-bisphosphatase 2 class 2	P21437|GLPX2_ECOLI	20
Transcription termination/antitermination protein NusA	P0AFF6|NUSA_ECOLI	20

*Proteins were isolated from CBD treated (1 μM) E. coli MVs and analyzed by LC-MS/MS. Peak list files were submitted to Mascot (in-house, Cambridge Center for Proteomics, Uniprot_Escherichia_coli_20180613; 4324 sequences; 1357163 residues)*.

‡ Ions score is −10^*^Log(P), where P is the probability that the observed match is a random event. Individual ions scores > 18 indicated identity or extensive homology (p < 0.05). Protein scores were derived from ions scores as a non-probabilistic basis for ranking protein hits.

**Table 3:** Proteins identified as present only in *E. coli* VCS257 derived MVs following 1 h treatment with 5 μM CBD.

**Protein name**	**Symbol**	**Score (*p* < 0.05)^‡^**
Glutamate decarboxylase alpha	P69908|DCEA_ECOLI	189
ATP-dependent zinc metalloprotease FtsH	P0AAI3|FTSH_ECOLI	128
Rod shape-determining protein MreB	P0A9X4|MREB_ECOLI	109
Uncharacterized protein YibN	P0AG27|YIBN_ECOLI	101
Outer membrane protein X	P0A917|OMPX_ECOLI	99
Galactitol 1-phosphate 5-dehydrogenase	P0A9S3|GATD_ECOLI	91
UPF0381 protein YfcZ	P0AD33|YFCZ_ECOLI	85
50S ribosomal protein L31	P0A7M9|RL31_ECOLI	83
Biotin carboxylase	P24182|ACCC_ECOLI	83
GMP synthase [glutamine-hydrolyzing]	P04079|GUAA_ECOLI	82
Cytochrome bd-I ubiquinol oxidase subunit 1	P0ABJ9|CYDA_ECOLI	74
Galactokinase	P0A6T3|GAL1_ECOLI	74
RNA chaperone ProQ	P45577|PROQ_ECOLI	71
Protein GrpE	P09372|GRPE_ECOLI	68
Purine nucleoside phosphorylase	P0ABP8|DEOD_ECOLI	61
50S ribosomal protein L21	P0AG48|RL21_ECOLI	59
Dihydrolipoyllysine-residue succinyltransferase component of 2-oxoglutarate dehydrogenase complex	P0AFG6|ODO2_ECOLI	58
Sec-independent protein translocase protein TatA	P69428|TATA_ECOLI	56
Bifunctional protein GlmU	P0ACC7|GLMU_ECOLI	56
PTS system mannose-specific EIIAB component	P69797|PTNAB_ECOLI	55
Anaerobic glycerol-3-phosphate dehydrogenase subunit C	P0A996|GLPC_ECOLI	54
Proline/betaine transporter	P0C0L7|PROP_ECOLI	52
Pyruvate formate-lyase 1-activating enzyme	P0A9N4|PFLA_ECOLI	52
Pyruvate/proton symporter BtsT	P39396|BTST_ECOLI	52
Protein translocase subunit SecY	P0AGA2|SECY_ECOLI	49
Penicillin-binding protein activator LpoB	P0AB38|LPOB_ECOLI	49
Signal peptidase I	P00803|LEP_ECOLI	45
Thiol peroxidase	P0A862|TPX_ECOLI	45
UPF0307 protein YjgA	P0A8X0|YJGA_ECOLI	45
Peptidyl-prolyl cis-trans isomerase D	P0ADY1|PPID_ECOLI	44
3-hydroxydecanoyl-[acyl-carrier-protein] dehydratase	P0A6Q3|FABA_ECOLI	44
ATP-dependent protease subunit HslV	P0A7B8|HSLV_ECOLI	43
Inosine-5'-monophosphate dehydrogenase	P0ADG7|IMDH_ECOLI	42
Peptide chain release factor RF2	P07012|RF2_ECOLI	41
Nucleoside diphosphate kinase	P0A763|NDK_ECOLI	40
Inositol-1-monophosphatase	P0ADG4|SUHB_ECOLI	40
Respiratory nitrate reductase 1 gamma chain	P11350|NARI_ECOLI	40
Succinate dehydrogenase hydrophobic membrane anchor subunit	P0AC44|DHSD_ECOLI	39
Outer membrane protein assembly factor BamB	P77774|BAMB_ECOLI	36
Signal recognition particle receptor FtsY	P10121|FTSY_ECOLI	36
Anaerobic C4-dicarboxylate transporter DcuB	P0ABN9|DCUB_ECOLI	34
Glucans biosynthesis protein	P33136|OPGG_ECOLI	34
Adenine phosphoribosyltransferase	P69503|APT_ECOLI	34
Maltoporin	P02943|LAMB_ECOLI	34
NADH-quinone oxidoreductase subunit C/D	P33599|NUOCD_ECOLI	32
ATP-dependent protease ATPase subunit HslU	P0A6H5|HSLU_ECOLI	32
CDP-diacylglycerol–serine O-phosphatidyltransferase	P23830|PSS_ECOLI	32
PTS system trehalose-specific EIIBC component	P36672|PTTBC_ECOLI	31
Transcription termination/antitermination protein NusG	P0AFG0|NUSG_ECOLI	31
Protein translocase subunit SecF	P0AG93|SECF_ECOLI	30
Oligopeptide transport system permease protein OppB	P0AFH2|OPPB_ECOLI	30
Uncharacterized protein YffS	P76550|YFFS_ECOLI	29
NADH-quinone oxidoreductase subunit J	P0AFE0|NUOJ_ECOLI	29
Glucosamine-6-phosphate deaminase	P0A759|NAGB_ECOLI	29
Uncharacterized protein YiaF	P0ADK0|YIAF_ECOLI	28
Tol-Pal system protein TolQ	P0ABU9|TOLQ_ECOLI	28
Multidrug export protein EmrA	P27303|EMRA_ECOLI	27
UPF0246 protein YaaA	P0A8I3|YAAA_ECOLI	25
DNA-directed RNA polymerase subunit omega	P0A800|RPOZ_ECOLI	24
ATP-binding/permease protein CydD	P29018|CYDD_ECOLI	24
Glycine betaine-binding protein YehZ	P33362|YEHZ_ECOLI	23
NADP-dependent malic enzyme	P76558|MAO2_ECOLI	23
Multiphosphoryl transfer protein	P69811|PTFAH_ECOLI	23
Ribose-5-phosphate isomerase A	P0A7Z0|RPIA_ECOLI	22
Disulfide bond formation protein B	P0A6M2|DSBB_ECOLI	22
Uncharacterized protein YbjD	P75828|YBJD_ECOLI	22
NADH-quinone oxidoreductase subunit L	P33607|NUOL_ECOLI	21
Pyridoxine 5'-phosphate synthase	P0A794|PDXJ_ECOLI	21

*Proteins were isolated from CBD treated (5 μM) E. coli MVs and analyzed by LC-MS/MS. Peak list files were submitted to Mascot (in-house, Cambridge Center for Proteomics, Uniprot_Escherichia_coli_20180613; 4324 sequences; 1357163 residues)*.

‡ Ions score is −10^*^Log(P), where P is the probability that the observed match is a random event. Individual ions scores > 19 indicated identity or extensive homology (p < 0.05). Protein scores were derived from ions scores as a non-probabilistic basis for ranking protein hits. Cut-off was set at Ions score 20.

## Discussion

To our knowledge this is the first study to evaluate the putative effects of CBD on the release of membrane vesicles (MVs) from bacteria and effects of CBD on MV profile, including protein composition. In eukaryotic cells, CBD was recently identified as an effective inhibitor of extracellular vesicle (EV) release both in human cancer cells (Kosgodage et al., 2018, 2019) as well as in the intestinal parasite *Giardia intestinalis* (Gavinho et al., 2019). Therefore, our present findings may indicate phylogenetically conserved pathways of membrane vesicle release from bacteria to mammals that can be modulated via CBD. Moreover, CBD could enhance the anti-bacterial effect of certain antibiotics in some bacterial types, but also inhibit it in others. This indicates that inhibition of MV release and anti-bacterial action are likely linked, as previously suggested (Tashiro et al., 2010). Indeed, a recent study using indole derivatives has revealed a role for MVs in antibiotic resistance/persistence, in particular in Gram-negative bacteria tested (Agarwal et al., 2019).

Here we report that CBD significantly reduced MV release in *E. coli* VCS257, a Gram-negative bacterium, but had negligible effects on membrane vesicle release in *S. aureus* subsp*. aureus* Rosenbach, a Gram-positive bacterium, as assessed here by *in vitro* analysis. In addition, we also found that lower doses of CBD had a stronger MV inhibitory effect in *E. coli* VCS257 than a higher 5 μM dose (*p* = 0.0063), and such an effect has also previously been observed for EVs in certain cancer cell types (Kosgodage et al., 2018). Biphasic effects of CBD are indeed recognized (Bergamaschi et al., 2011) and may be reminiscent of “hormesis,” an effect we have suggested could explain its more general medical benefits as well as effects on mitochondrial dynamics (Nunn et al., 2013). Interestingly, at the lower 1 μM concentration, CBD significantly increased the release of a 500 nm peak of MVs, as observed by NTA analysis, while this peak was negligible both in the control treated bacteria and those treated with 5 μM CBD. Such an effect of CBD on MV profile, and protein MV profile as observed by proteomic analysis here, may be relevant in the light of recent recognition of the importance of MV size for cellular entry and uptake (Turner et al., 2018) and in line with an increased interest in the research community for the identification and characterization of MV sub-populations (Pérez-Cruz et al., 2016; Turner et al., 2018; Cooke et al., 2019; Toyofuku et al., 2019; Zavan et al., 2019). The approximately 6.5-fold and 2.5-fold decreases in MV release observed after CBD (1 and 5 μm, respectively) treatment from *E. coli*, compared to non-treated controls, also correlated with a trend in shift toward proportionally larger vesicles released according to NTA analysis and change in protein profile. The exact mechanism for packaging proteins and other reagents in MVs is not fully understood and given the plethora of targets for CBD (Ibeas Bih et al., 2015; Hernández-Cervantes et al., 2017; Pisanti et al., 2017), the exact mechanism of this cannabinoid on MV formation remains subject to further extensive studies. In the current study we have indeed identified a range proteins, including proteins involved in metabolism and antibiotic metabolic processing, which differ in MVs released from *E. coli* VCS257 treated with CBD, compared to MVs released from non-treated *E. coli*. Previous studies have discussed the use of MVs for example as drug delivery vehicles (Ellis and Kuehn, 2010; Gujrati et al., 2014; Gerritzen et al., 2017; Jain and Pillai, 2017; Jan, 2017; Wang et al., 2018), while MVs have also been tested as delivery vehicles for targeted gene silencing using siRNA-packaged MVs (Alves et al., 2016). Whether CBD may be utilized for combinatory application with such approaches may also be of putative interest, in addition to its observed effects in this study, in effectively reducing MV release.

In relation to antibiotic activity, cannabinoids including CBD, have been widely studied for their anti-bacterial activity (Wasim et al., 1995; Bass et al., 1996; Appendino et al., 2008; Hernández-Cervantes et al., 2017). For example, *C. sativa* extracts have previously been shown to have microbicidal activity on various Gram-positive bacteria, including several strains of *S. aureus*, as well as some Gram-negative bacteria (Wasim et al., 1995; Elphick, 2007; Nissen et al., 2010), with the minimum inhibitory concentrations (MIC) for the main phytocannabinoids, such as CBD, being in the 0.5–5 μM range, which is similar to many modern antibiotics (Van Klingeren and Ten Ham, 1976; Appendino et al., 2008). How precisely CBD may be working as an anti-bacterial agent is still not entirely clear (Appendino et al., 2008), particularly in the light of a plethora of targets for CBD (Ibeas Bih et al., 2015; Hernández-Cervantes et al., 2017), while structure-activity studies indicate that the ability of plant-derived phenolic compounds to interact with membranes and the existence of electrophilic functional groups are important (Miklasinska-Majdanik et al., 2018). Hitherto though, no association has been made into a putative regulatory effect of cannabinoids on bacterial membrane vesicle release. Furthermore, as the current study has revealed changes in proteomic profile of MVs released from *E. coli* VCS257 following CBD treatment, such findings may inform anti-bacterial effects of CBD. Using LC-MS/MS analysis to assess changes in protein profile of MVs from CBD treated and untreated *E. coli*, respectively, five proteins were found to be absent in the 1 μM CBD treated MVs and 4 proteins were absent in the 5 μM CBD treated MVs, compared to control untreated *E. coli* MVs. Out of these, 2 proteins overlapped between the two CBD treatments. In addition, comparing 1 and 5 μM CBD treated *E. coli* MVs, 26 protein hits were unique to MVs released following the 1 μM CBD treatment and 68 protein hits to MVs released following the 5 μM CBD treatment. Using STRING analysis, PPI enrichment *p*-value was found to be *p* = 0.0204 for proteins identified as unique to MVs from the 1 μM CBD treatment and *p* = 1.56 × 10^−6^ for proteins identified as unique to MVs from the 5 μM CBD. This indicates that for both treatments these proteins have significantly more interactions among themselves, than what would be expected for a random set of proteins of similar size, drawn from the genome. Such enrichment indicates that the proteins are at least partially biologically connected, as a group. Protein networks are represented showing biological GO pathways and KEGG pathways, respectively, in Supplementary Figures 3, 4 for proteins specific to EVs from *E. coli* after 1 and 5 μM CBD treatment, respectively. Proteins identified are related to metabolic processes, cellular respiration and antibiotic functions (Supplementary Figures 3A,B, 4A,B).

When assessing the effectivity of CBD to enhance susceptibility of Gram-positive and Gram-negative bacterial species to a range of antibiotics, CBD-mediated MV inhibition rendered *E. coli* VCS257 significantly more sensitive to erythromycin, vancomycin and rifampicin and somewhat to kanamycin, but did not augment the bactericidal effects observed for colistin. This was somewhat unexpected, given a previous study showing that MVs isolated from the *E. coli* strain MG1655 could protect bacteria against membrane-active antibiotics such as colistin (Kulkarni et al., 2015). Our finding, that CBD did not sensitize *E. coli* further to colistin, when applied in combination with this antibiotic, may arise from the fact that a different strain of *E. coli* (VCS257) was used in the current study, compared to in the study by Kulkarni et al. (2015). It has also been previously shown that the presence of calcium decreases the bactericidal effect of colistin on *Paenibacillus polymyxa*, suggesting a role for Ca^2+^ in generating a protective barrier against colistin (Yu et al., 2015). As CBD is known to modulate calcium (Rimmerman et al., 2013) it can be postulated that this may interfere with the mode of action of colistin. Our findings also indicate that combinatory application of CBD is not effective for all antibiotics, which may possibly be explained by their different modes of action. Importantly, zones of inhibition were observed in the plates which were only treated with the CBD discs in the presence of *E. coli*, and this clearly revealed the antibacterial property of CBD.

Interestingly, CBD did increase antibacterial effects of vancomycin on *E. coli*, in spite of vancomycin's limited effectiveness on Gram-negative species, also seen here by the fact that vancomycin alone did not result in a halo around the diffusion disk for *E. coli*. Therefore, CBD seems to overcome previously established resistance of *E. coli* to vancomycin, which has reported to partly be due to its inability to significantly penetrate the outer membrane (Zhou et al., 2015). It may also be important to note that erythromycin, rifampicin and kanamycin inhibit protein synthesis, whereas vancomycin is a glycopeptide that inhibits cell biosynthesis in Gram-positive bacteria, while colistin binds to the outer membrane of Gram-negative bacteria, disrupting it. Thus, these antibiotics display very different modes of action.

In the Gram-positive bacterium *S. aureus* subsp. *aureus* Rosenbach, CBD increased bactericidal activity of kanamycin only. The reduced ability of CBD to sensitize this Gram-positive bacterium to antibiotics, compared to the significantly higher effects in the Gram-negative bacterium, tallied in with CBD's ability to regulate MV-release, indicating a relevant contribution of MVs to antibiotic resistance. Roles for MVs in protecting biofilms via adsorption of antimicrobial agents have indeed been previously recognized (Schooling and Beveridge, 2006; Manning and Kuehn, 2011; Toyofuku et al., 2019). This also indicates that MV-inhibitors that target membrane vesicles from specific bacteria species, such as CBD here, could be applied in combination with selected antibiotics for tailored antibiotic treatment to tackle antibiotic resistance.

## Conclusions

CBD effectively inhibited MV release from the Gram-negative bacterium *E. coli* VCS257, exhibiting a stronger MV-inhibiting effect at lower dose. In addition, CBD modulated MV protein profiles of *E. coli* following 1 h treatment. CBD did not have significant effects on MV release in the Gram-positive bacterium *S. aureus* subsp*. aureus* Rosenbach. When applied in combination with a range of antibiotics, CBD increased anti-bacterial effects of selected antibiotics, depending on bacteria type. CBD, in combination with specific antibiotics, may thus possibly be used as an adjuvant to selectively target bacteria to sensitize them to antibiotic treatment and reduce antibiotic resistance.

## Data Availability

All datasets generated for this study are included in the manuscript and/or the Supplementary Files.

## Author Contributions

UK, PM, BA, IK, PW, and SL performed the experiments. UK, JB, AN, JI, and SL analyzed the data. PM, GM, GG, IK, SL, and JI provided resources. UK, SL, and JI designed the study. SL, UK, and AN wrote the manuscript. All authors critically reviewed the manuscript.

### Conflict of Interest Statement

GG is founder and chairman of GW Pharmaceuticals. AN is a scientific advisor to GW Pharmaceuticals. The remaining authors declare that the research was conducted in the absence of any commercial or financial relationships that could be construed as a potential conflict of interest.

## Supplementary Material

Supplementary Figure 1 Effects of CBD on bacterial growth in **(A)** *E. coli* VCS257 and **(B)** *S. aureus* subsp. *aureus* Rosenbach, after 24 h incubation, as assessed by disk diffusion test. Exact *p*-values are shown.

Supplementary Figure 2 TEM of MV released from *E. coli* VCS257 following 1 or 5 μM CBD treated for 1 h, compared to MVs isolated from control, untreated *E. coli*. **(A)** A composite image showing MVs released from control, untreated *E. coli*. **(B)** A composite image showing MVs released from *E. coli* treated with 1 μM CBD for 1 h. **(C)** A composite image showing MVs released from *E. coli* treated with 5 μM CBD for 1 h. Scale bars indicate 100 nm, respectively, and are included in the individual figures.

Supplementary Figure 3 Protein-protein interaction networks of protein hits identified in MVs from 1 μM CBD treated *E. coli* VCS257. Reconstruction of protein-protein interactions based on known and predicted interactions using STRING analysis. Colored nodes represent query proteins and first shell of interactors; white nodes are second shell of interactors. **(A)** Biological GO processes are highlighted as follows: red, citrate metabolic process; green, antibiotic metabolic process; yellow, regulation of cellular amide metabolic process; purple, carboxylic acid metabolic process; dark green, regulation of phosphate metabolic process; light blue, cellular respiration; orange, small molecule metabolic process; dark red, negative regulation of translational elongation; dark blue, generation of precursor metabolites and energy. **(B)** KEGG pathways are highlighted as follows: dark green, oxidative phosphorylation; dark red, citrate cycle (TCA cycle); red, biosynthesis of antibiotics; purple, butanoate metabolism; dark blue, biosynthesis of secondary metabolites; light blue, carbon metabolism; orange, phenylalanine, tyrosine and tryptophan biosynthesis; light green, Microbial metabolism in diverse environments; yellow, Metabolic pathways; violet, glycine, serine, and threonine metabolism. Colored lines indicate whether protein interactions are identified via known interactions (curated databases, experimentally determined), predicted interactions (gene neighborhood, gene fusion, gene co-occurrence) or via text mining, co-expression or protein homology (see color key for connective lines).

Supplementary Figure 4 Protein-protein interaction networks of protein hits identified in MVs from 5 μM CBD treated *E. coli* VCS257. Reconstruction of protein-protein interactions based on known and predicted interactions using STRING analysis. Colored nodes represent query proteins and first shell of interactors; white nodes are second shell of interactors. **(A)** Biological GO processes are highlighted as follows: red, cellular respiration; green, purine-containing compound metabolic process; yellow, electron transport chain; purple, ribose phosphate metabolic process; dark green, purine ribonucleotide metabolic process; light blue, generation of precursor metabolites and energy; orange, nucleobase-containing small molecule metabolic process; dark red, purine ribonucleoside metabolic process; dark blue, organophosphate metabolic process. **(B)** KEGG pathways are highlighted as follows: red, bacterial secretion system; light green, metabolic pathways; yellow, oxidative phosphorylation; purple, butanoate metabolism; dark green, quorum sensing; light blue, amino sugar and nucelotide sugar metabolism; dark blue, protein export; violet, purine metabolism. Colored lines indicate whether protein interactions are identified via known interactions (curated databases, experimentally determined), predicted interactions (gene neighborhood, gene fusion, gene co-occurrence) or via text mining, co-expression or protein homology (see color key for connective lines).
