# Supplementary figures and images for: Cannabidiol Is a Novel Modulator of Bacterial Membrane Vesicles
Source: Front Cell Infect Microbiol. 2019 Sep 10;9:324. doi: 10.3389/fcimb.2019.00324 (PMC6747004; doi:10.3389/fcimb.2019.00324)

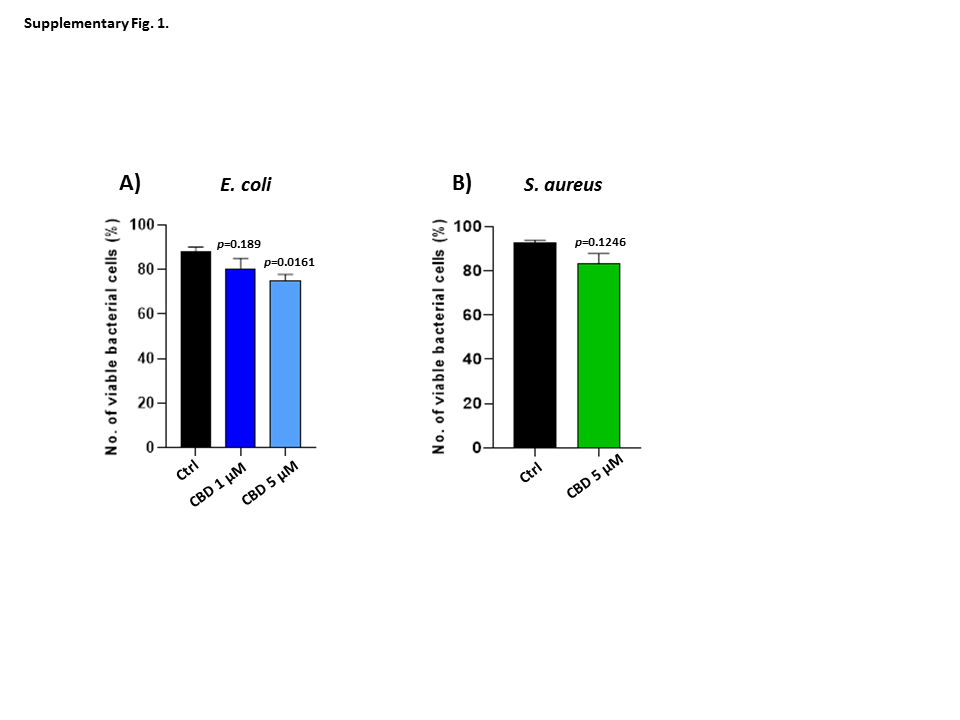

Supplement: Supplementary Figure 1 — Effects of CBD on bacterial growth in (A) E. coli VCS257 and (B) S. aureus subsp. aureus Rosenbach, after 24 h incubation, as assessed by disk diffusion test. Exact p-values are shown. [file Image_1.TIF]

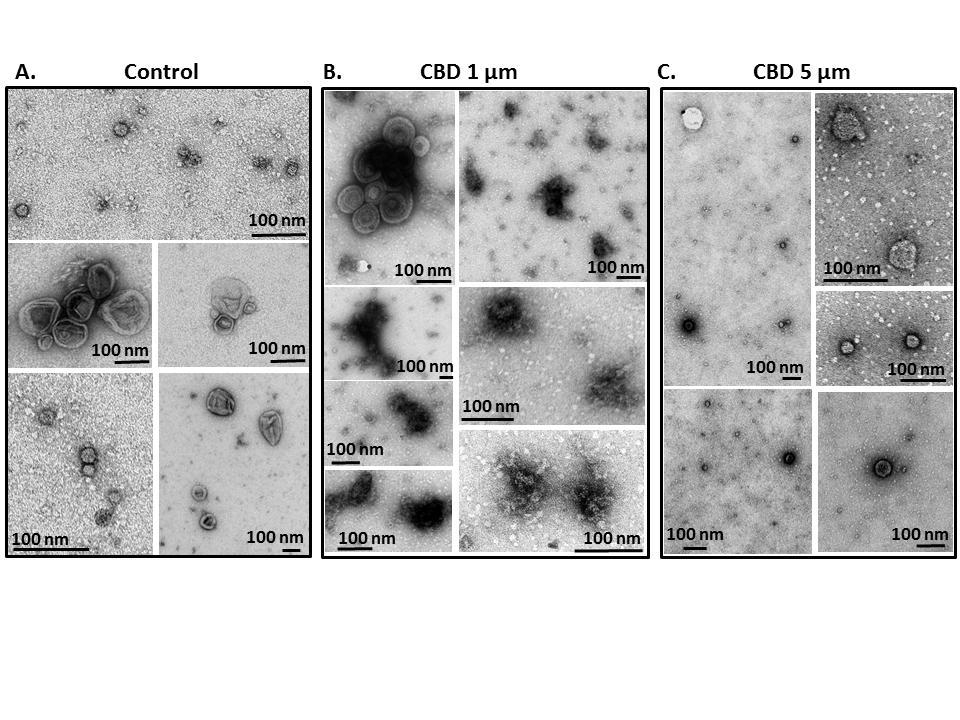

Supplement: Supplementary Figure 2 — TEM of MV released from E. coli VCS257 following 1 or 5 μM CBD treated for 1 h, compared to MVs isolated from control, untreated E. coli. (A) A composite image showing MVs released from control, untreated E. coli. (B) A composite image showing MVs released from E. coli treated with 1 μM CBD for 1 h. (C) A composite image showing MVs released from E. coli treated with 5 μM CBD for 1 h. Scale bars indicate 100 nm, respectively, and are included in the individual figures. [file Image_2.TIF]

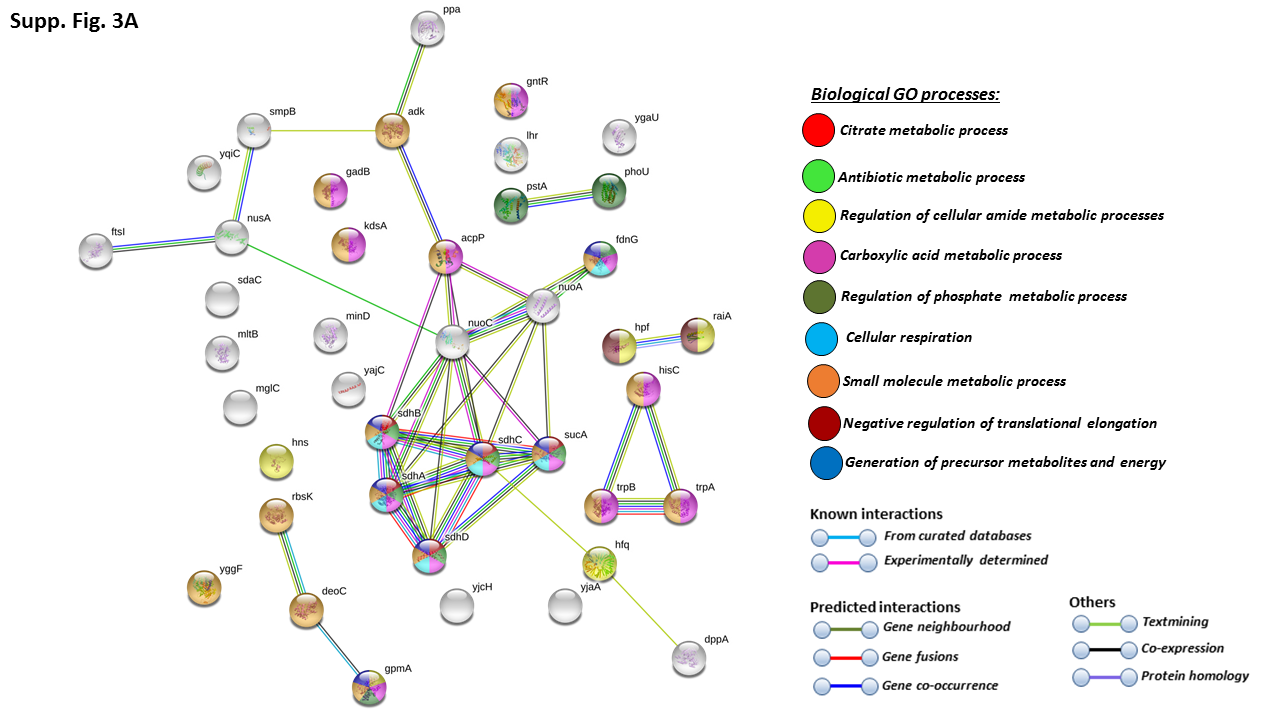

Supplement: Supplementary Figure 3 — Protein-protein interaction networks of protein hits identified in MVs from 1 μM CBD treated E. coli VCS257. Reconstruction of protein-protein interactions based on known and predicted interactions using STRING analysis. Colored nodes represent query proteins and first shell of interactors; white nodes are second shell of interactors. (A) Biological GO processes are highlighted as follows: red, citrate metabolic process; green, antibiotic metabolic process; yellow, regulation of cellular amide metabolic process; purple, carboxylic acid metabolic process; dark green, regulation of phosphate metabolic process; light blue, cellular respiration; orange, small molecule metabolic process; dark red, negative regulation of translational elongation; dark blue, generation of precursor metabolites and energy. (B) KEGG pathways are highlighted as follows: dark green, oxidative phosphorylation; dark red, citrate cycle (TCA cycle); red, biosynthesis of antibiotics; purple, butanoate metabolism; dark blue, biosynthesis of secondary metabolites; light blue, carbon metabolism; orange, phenylalanine, tyrosine and tryptophan biosynthesis; light green, Microbial metabolism in diverse environments; yellow, Metabolic pathways; violet, glycine, serine, and threonine metabolism. Colored lines indicate whether protein interactions are identified via known interactions (curated databases, experimentally determined), predicted interactions (gene neighborhood, gene fusion, gene co-occurrence) or via text mining, co-expression or protein homology (see color key for connective lines). [file Image_3.TIF]

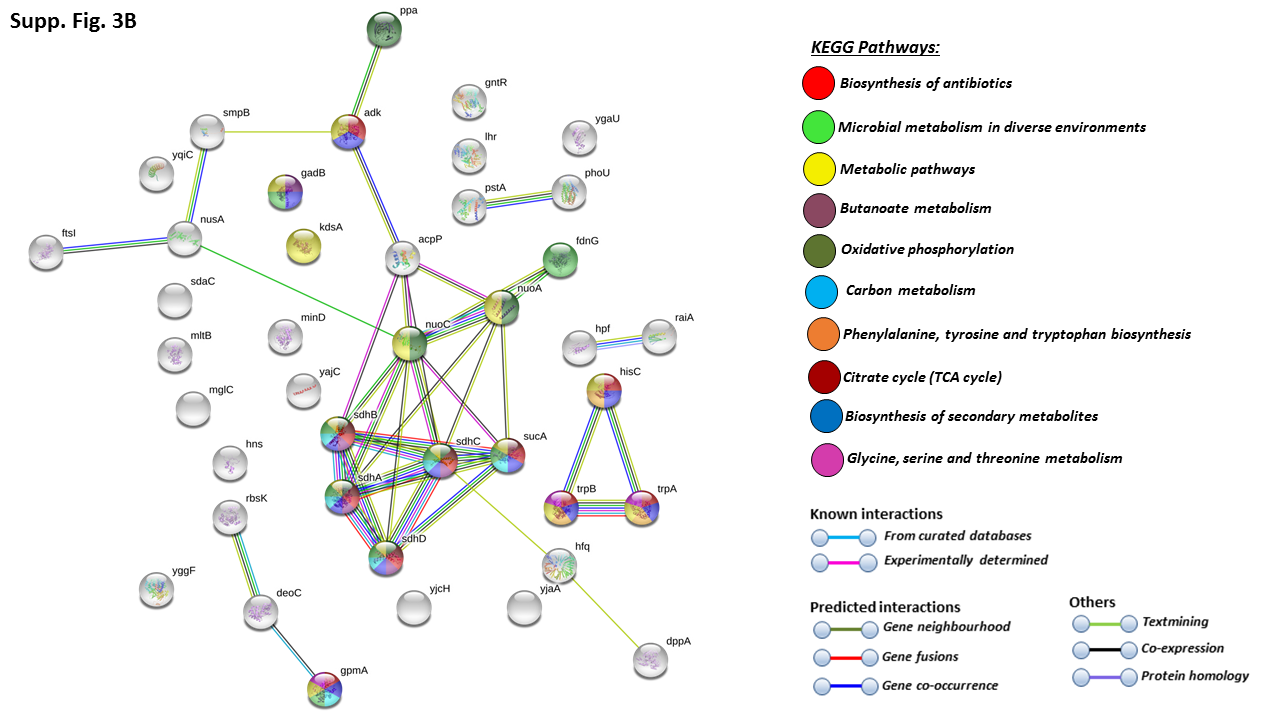

Supplement: Supplementary Figure 4 — Protein-protein interaction networks of protein hits identified in MVs from 5 μM CBD treated E. coli VCS257. Reconstruction of protein-protein interactions based on known and predicted interactions using STRING analysis. Colored nodes represent query proteins and first shell of interactors; white nodes are second shell of interactors. (A) Biological GO processes are highlighted as follows: red, cellular respiration; green, purine-containing compound metabolic process; yellow, electron transport chain; purple, ribose phosphate metabolic process; dark green, purine ribonucleotide metabolic process; light blue, generation of precursor metabolites and energy; orange, nucleobase-containing small molecule metabolic process; dark red, purine ribonucleoside metabolic process; dark blue, organophosphate metabolic process. (B) KEGG pathways are highlighted as follows: red, bacterial secretion system; light green, metabolic pathways; yellow, oxidative phosphorylation; purple, butanoate metabolism; dark green, quorum sensing; light blue, amino sugar and nucelotide sugar metabolism; dark blue, protein export; violet, purine metabolism. Colored lines indicate whether protein interactions are identified via known interactions (curated databases, experimentally determined), predicted interactions (gene neighborhood, gene fusion, gene co-occurrence) or via text mining, co-expression or protein homology (see color key for connective lines). [file Image_4.TIF]

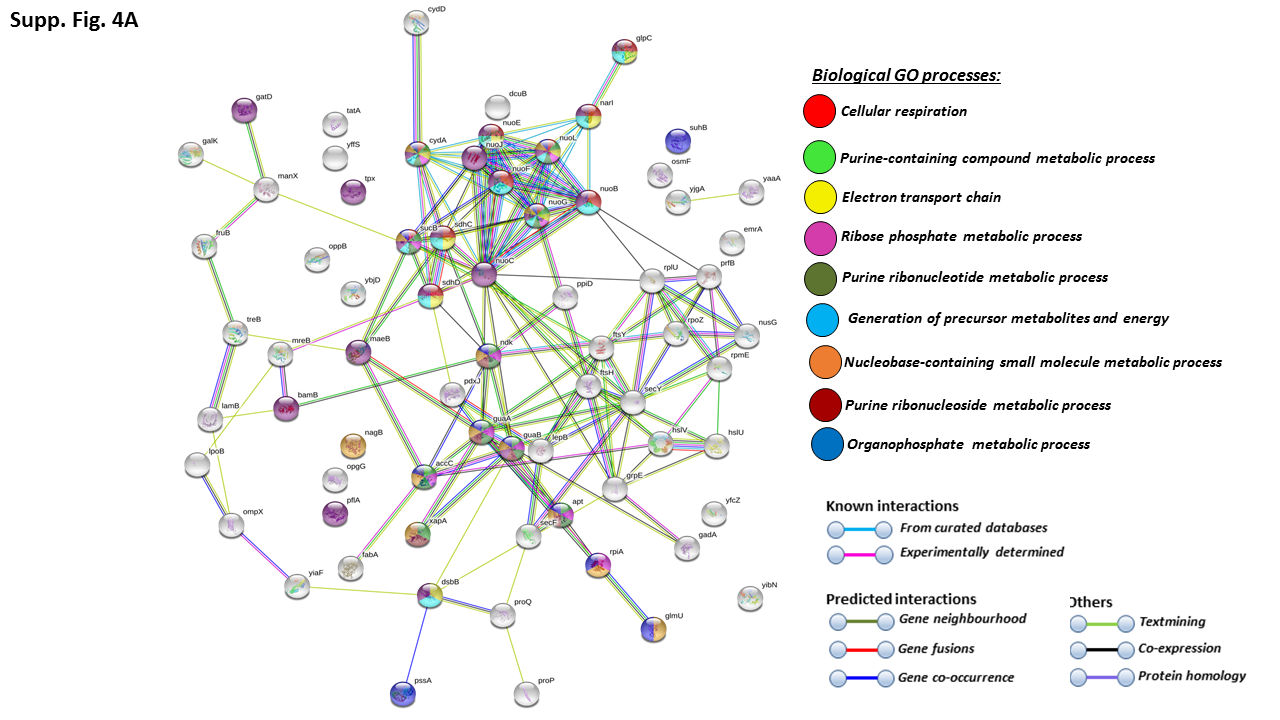

Supplement: Supplementary file 5 [file Image_5.TIF]

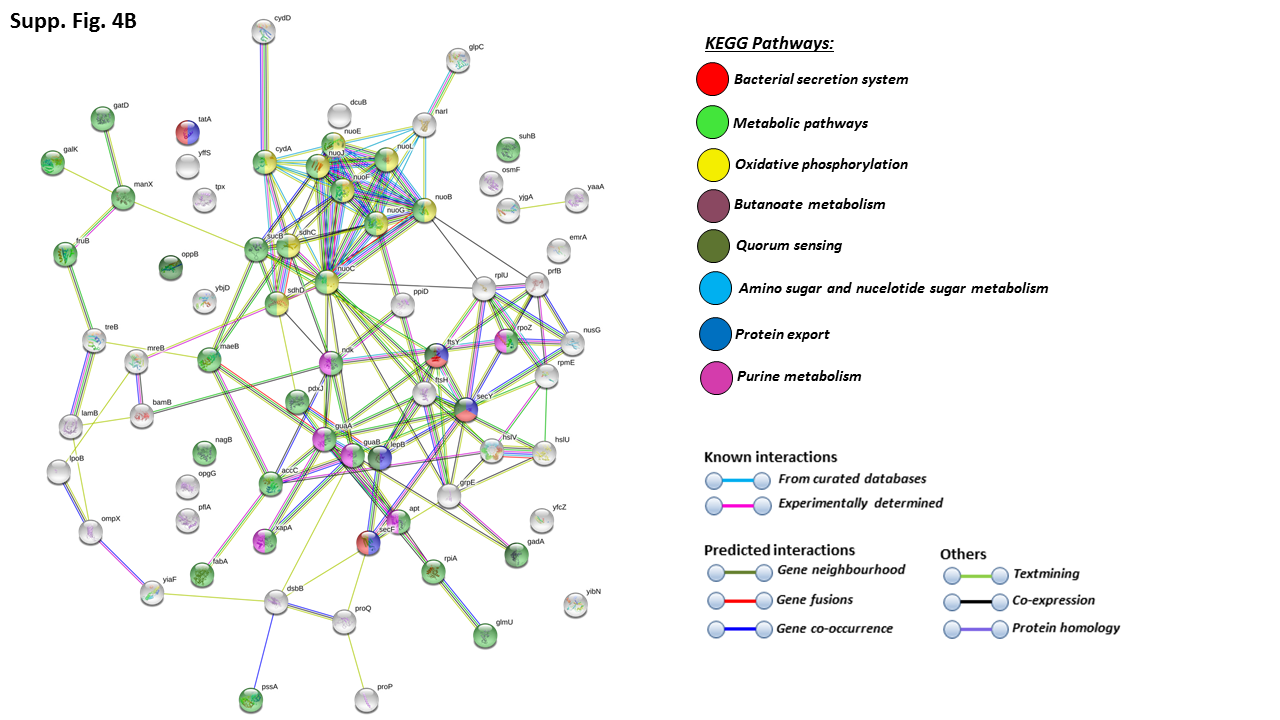

Supplement: Supplementary file 6 [file Image_6.TIF]
